# Ultrasonographic Ocular Biometry of the Greater Caribbean Manatee (*Trichechus manatus manatus*)

**DOI:** 10.1111/vop.70126

**Published:** 2025-11-28

**Authors:** Radan Elvis Matias de Oliveira, Fernanda Loffler Niemeyer Attademo, Rita de Kássia Matias de Oliveira, Romário Parente dos Santos, Fábia de Oliveira Luna, Antonio A. Mignucci‐Giannoni, Lesly J. Cabrias‐Contreras, Fabrício Bezerra de Sá, Flávio José de Lima Silva, Augusto Carlos da Bôaviagem Freire, Moacir Franco de Oliveira

**Affiliations:** ^1^ Programa de Pós‐Graduação Em Ciência Animal, Universidade Federal Rural Do Semi‐Árido Mossoró RN Brazil; ^2^ Programa de Pós‐Graduação Em Biologia Estrutural e Funcional, Departamento de Morfologia, Centro de Biociências Universidade Federal Do Rio Grande Do Norte Natal RN Brazil; ^3^ Centro de Estudos e Monitoramento Ambiental Areia Branca RN Brazil; ^4^ Instituto Chico Mendes de Conservação da Biodiversidade/ICMBio, Centro Nacional de Pesquisa e Conservação de Mamíferos Aquáticos/CMA Ilha de Itamaracá/PE Brazil; ^5^ Universidade Federal da Paraíba Areia Paraíba Brazil; ^6^ Caribbean Manatee Conservation Center Interamericana American University of Puerto Rico Bayamón Puerto Rico; ^7^ Center for Conservation Medicine and Ecosystem Health, Biomedical Sciences, Ross University School of Veterinary Medicine Basseterre St. Kitts and Nevis; ^8^ Universidade Federal Rural de Pernambuco Recife Pernambuco Brazil; ^9^ Projeto Cetáceos da Costa Branca, Universidade Do Estado Do Rio Grande Do Norte Mossoró RN Brazil; ^10^ Programa de Doutorado Em Desenvolvimento e Meio Ambiente Universidade Federal Do Rio Grande Do Norte Natal RN Brazil

**Keywords:** cornea, diagnostic imaging, eye, lens, ophthalmology, Sirenia

## Abstract

This study aimed to characterize the ocular biometry of the *Trichechus manatus manatus* applying B‐mode ultrasonography across different age groups. Twenty‐two animals were assessed employing a portable ultrasound device equipped with a linear transducer. Five ocular parameters were assessed: corneal thickness (CT), anterior chamber depth (ACD), axial lens length (ALL), vitreous chamber depth (VCD), and axial globe length (AGL). Data were statistically analyzed at a 5% significance level based on normality and variance. The mean values (cm) for the right eye of calves, juveniles, and adults were, respectively, CT (0.082 ± 0.012; 0.088 ± 0.011; 0.091 ± 0.010), ACD (0.040 ± 0.005; 0.051 ± 0.008; 0.052 ± 0.007), ALL (0.345 ± 0.028; 0.367 ± 0.016; 0.414 ± 0.020), VCD (1.137 ± 0.076; 1.155 ± 0.062; 1.161 ± 0.089), and AGL (1.606 ± 0.098; 1.663 ± 0.071; 1.720 ± 0.094). Values for the left eye were CT (0.082 ± 0.012; 0.088 ± 0.012; 0.087 ± 0.011), ACD (0.041 ± 0.008; 0.048 ± 0.006; 0.052 ± 0.007), ALL (0.345 ± 0.028; 0.381 ± 0.027; 0.411 ± 0.025), VCD (1.147 ± 0.076; 1.174 ± 0.045; 1.166 ± 0.086), and AGL (1.617 ± 0.086; 1.693 ± 0.062; 1.717 ± 0.095). CT, VCD, and AGL were similar across age groups, while ACD and ALL increased with age. Positive correlations between body length and ocular parameters were significant only in calves. Ocular ultrasonography is, thus, an effective, safe, and reproducible tool for ophthalmic evaluations in *T. m. manatus*.

## 1 Introduction

The order *Sirenia* comprises the only fully aquatic herbivorous mammals on the planet and has continuously faced numerous challenges due to anthropogenic factors. This has led to a decline in their original populations, primarily due to past hunting and ongoing habitat loss and calf stranding [1, 2]. The distribution of the American manatee ( *Trichechus manatus* ) is limited to part of the western North and South Atlantic coast, with two recognized subspecies, namely the Florida manatee (*T. m. latirostris*), found in the Southeastern United States, and the Greater Caribbean manatee (*T. m. manatus*) [3], distributed throughout the Caribbean and Central and South America [4].

*Trichechus m. manatus* is currently classified as “Vulnerable” globally by the International Union for Conservation of Nature [5], and “Endangered” in Brazil [6]. The current conservation scenario combined with certain biological characteristics raises concerns regarding the future of the *T. m. manatus* subspecies in Brazil [7, 8]. To minimize ecological impacts caused by stranding events, the Brazilian government, along with specialized conservation institutions, such as non‐governmental organizations and universities, conducts continuous rescue and rehabilitation efforts concerning stranded manatees, aiming to release them back into their natural habitats [1, 9]. Individuals under human care are valuable regarding behavior, health studies and, especially, concerning the standardization of diagnostic imaging techniques like ultrasonography, radiography, and computed tomography. The data obtained from these approaches not only benefit animals under human care but also expand knowledge on manatees in the wild.

Sonographic studies in manatees have been poorly addressed to date, with only one report focusing exclusively on certain digestive system structures in Amazonian manatees ( *Trichechus inunguis* ) [10]. Ocular structures, on the other hand, have not yet been assessed. In this sense, one of the main clinical medicine gaps and challenges concerning manatees corresponds to recently described ophthalmological problems [11, 12]. The enophthalmic eye position, large body size, and unique evolutionary adaptations of manatees have posed challenges to the standardization of diagnostic tools, particularly when compared to those successfully implemented in other aquatic mammals [13, 14]. In this context, implementing diagnostic imaging, i.e., sonography, could represent a promising alternative for clinical‐ophthalmological manatee evaluations.

Ocular ultrasonography is a non‐invasive method that enables the evaluation of various ocular structures, providing accurate diagnoses concerning the numerous conditions that affect the eye and its adnexa, such as cataracts, glaucoma, retinal detachment, intraocular tumors, intraocular and orbital hemorrhages, intraocular foreign bodies, corneal ulcers, and exophthalmos [15, 16, 17, 18, 19, 20, 21]. This technique is noteworthy in enabling the visualization of intraocular structures, such as the lens, vitreous chamber, and retina. This allows for more precise diagnoses and monitoring of ophthalmic changes often unnoticed during conventional clinical examinations, which are generally limited to the cornea, nictitating membrane, and light reflex assessments. However, before investigating pathological changes through ultrasonography, baseline ultrasonographic parameters should be established for different species. Significant gaps, however, are noted for ocular ultrasonographic standards for sirenians, highlighting the need for further studies to support the clinical and conservation management of these animals in captivity.

Considering the importance of manatees, several conservation strategies have been proposed to promote wild population stabilization [7, 8, 22, 23]. Accordingly, this study aimed to characterize the B‐mode ocular ultrasonographic biometry of the Greater Caribbean manatee eye across different age groups, providing novel information concerning the ophthalmic care of these animals, as establishing ocular ultrasonographic patterns allows for the generation of scientific data that may improve the diagnosis of various eye diseases.

## 2 Material and Methods

### 2.1 Animals

Ocular ultrasonographic feature assessments were carried out for 22 Greater Caribbean manatees, both males and females and consisting of various age groups (calves, juveniles, and adults) (Table 1). The determination of age/age group and body weight of the manatees evaluated herein was carried out according to Attademo et al. [24]. All individuals stranded during the neonatal phase presented an umbilical cord, indicating that stranding occurred within the first week of life. For classification purposes, manatees aged 0–24 months were considered calves, those aged 25–72 months, juveniles, and individuals older than 72 months, adults. Body weight was estimated using a validated mathematical formula based on biometric measurements.

**TABLE 1:** Greater Caribbean manatee individuals ( *Trichechus manatus manatus* ) evaluated in this study concerning ocular ultrasonographic biometry characterizations.

Individuals	Sex	Age	Age group	BW	STL
Animal 1	Male	5m	Calf	37	110
Animal 2	Male	8m	Calf	89.5	171
Animal 3	Male	1y/2m	Calf	186.6	211
Animal 4	Male	1y/4m	Calf	95	154
Animal 5	Male	1y/6m	Calf	167.2	202
Animal 6	Female	1y/2m	Calf	105	165
Animal 7	Female	1y/7m	Calf	197.7	219
Animal 8	Male	2y/7m	Juvenile	237.6	231
Animal 9	Male	2y/7m	Juvenile	204	222
Animal 10	Male	3y/5m	Juvenile	278.6	241.4
Animal 11	Male	3y/8m	Juvenile	337.8	248.2
Animal 12	Female	2y/6m	Juvenile	266.4	242.6
Animal 13	Female	2y/7m	Juvenile	267.7	236.2
Animal 14	Female	3y/3m	Juvenile	268.3	244
Animal 15	Female	3y/8m	Juvenile	336.3	244
Animal 16	Male	6y/1m	Adult	317.2	244
Animal 17	Male	32y	Adult	365	254
Animal 18	Female	6y/1m	Adult	448.5	284.2
Animal 19	Female	7y/4m	Adult	206	232
Animal 20	Female	12y	Adult	528	297
Animal 21	Female	28y	Adult	558	312
Animal 22	Female	28y	Adult	524	300

Abbreviations: BW, body weight (kg); m, months; STL, straight total length (cm); y, years.

The study was conducted on Greater Caribbean manatees undergoing rehabilitation under the care of the Cetacean Project of the Costa Branca at the State University of Rio Grande do Norte (PCCB‐UERN), located in the municipality of Areia Branca, Rio Grande do Norte, Brazil (4°55′43″S, 37°7′58″W) and the National Center for Research and Conservation of Aquatic Mammals—Chico Mendes Institute for Biodiversity Conservation (CMA/ICMBio), located in the municipality of Ilha de Itamaracá, Pernambuco, Brazil (7°48′33″S, 34°50′19″W). Ocular ultrasonographic examinations were performed during routine clinical management procedures, which are carried out monthly at the rehabilitation centers involved in this study. These procedures are part of the regular activities developed by the institutions responsible for the rescue, rehabilitation, and release of marine animals, with a focus on biodiversity conservation and the assessment of human impact on aquatic fauna. During these procedures, clinical evaluations, biological sampling (including blood, feces, urine, and oral, genital, and anal swabs), and body biometric assessments are performed to monitor the health and development of the rehabilitating animals. Due to the larger sizes and body weights of juvenile and adult individuals, the pool was drained, and examinations were conducted inside the pool, allowing for safe and adequate assessments.

Ocular ultrasonography was integrated into these procedures in a non‐invasive manner, ensuring the safety and well‐being of the animals throughout the examinations. All activities carried out by both institutions were properly authorized by the relevant environmental agencies, according to permits issued by the Biodiversity Authorization and Information System (SISBIO), under permit numbers 13694‐12 for manatees at PCCB‐UERN and 20 685‐9 for manatees at CMA/ICMBio. All animal handling and procedures were conducted in accordance with animal welfare guidelines and approved by the Animal Ethics Committee of the Federal University of the Semi‐Arid Region, under approval number 31/2024. Therefore, the study followed strict and ethical protocols to ensure the welfare of the animals during rehabilitation.

### 2.2 Ocular Ultrasonography

The animals were manually restrained by the veterinary team and animal caretakers from each institution, following strict protocols to ensure animal welfare without the use of sedatives. Individual measurements, including body weight (BW) and straight total length (STL), were also recorded for each specimen prior to the ultrasonographic evaluations.

The ophthalmic examination was performed in all conscious animals prior to ocular ultrasonography, with the aim of identifying any abnormalities. Each eye was evaluated using a slit‐lamp biomicroscope (Keeler PSL Classic; Keeler Ltd., Windsor, UK) and a direct ophthalmoscope (HEINE BETA K 180, DE). The neuro‐ophthalmic assessment indicates a menace response with an intact dazzle reflex. The cornea was clear and healthy, without opacities, defects, or fluorescein uptake. No aqueous flare or signs of anterior segment inflammation were observed. Pupils were round with no opacifications found in the lens or its anterior and posterior capsules. In the posterior segment, the vitreous was transparent; however, a detailed visualization of the posterior pole was limited due to the animals' agitated behavior and rapid eyelid‐closing reflex.

**FIGURE 1:**
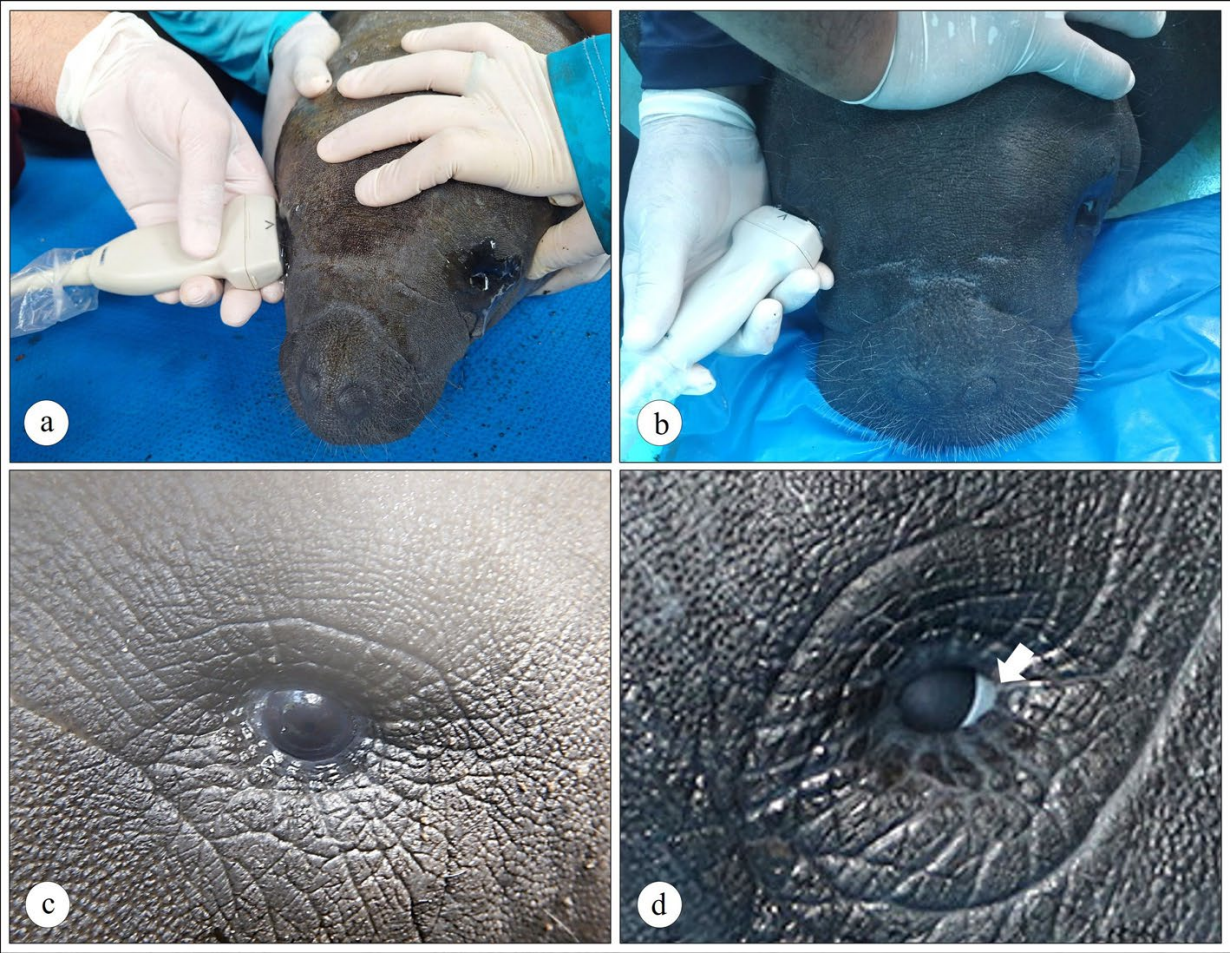
B‐mode ocular ultrasound of Greater Caribbean manatees ( *Trichechus manatus manatus* ). (a) B‐mode scan of the right eye of a manatee calf. (b) B‐mode scan of the right eye of a juvenile manatee. (c) Left eye. (d) Right eye nictitating membrane (arrow).

One drop of topical anesthetic (1% tetracaine hydrochloride containing 0.1% phenylephrine, Anestésico, Allergan, São Paulo, Brazil) was administered to each eye just prior to the ultrasonographic assessments. This dose was sufficient to allow for the ultrasonographic examinations to be performed for up to 20 min. A portable Mindray Z50 ultrasound system (Shenzhen Mindray Bio‐Medical Electronics CO., Shenzhen, China) equipped with a 7–12 MHz linear transducer was employed. An odorless and colorless lubricant gel (KY) was applied both to the corneal surface and to the transducer, followed by gently placing the transducer on the cornea of the examined manatees. B‐mode ocular scanning was performed in axial, horizontal, or oblique sections by the same examiner to minimize inter‐examiner measurement errors (Figure 1a,b). It is important to note that the Greater Caribbean manatee has circularly shaped eyelids that close through a muscle sphincter‐like mechanism, in addition to a nictitating membrane (Figure 1c,d), which can make ophthalmic examinations more difficult, especially when the animal is stressed during physical restraint.

To overcome the challenge of eyelid closure caused by the sphincter‐like mechanism of manatee eyelids, a considerable layer of lubricant gel was applied both to the corneal surface and to the transducer. The probe was gently positioned near the eye, without touching the corneal surface, maintaining the gel layer as an acoustic interface to mimic the aqueous environment. The examiner then waited for the animals to spontaneously open their eyes, enabling the acquisition of clear transcorneal ultrasonographic images.

**FIGURE 2:**
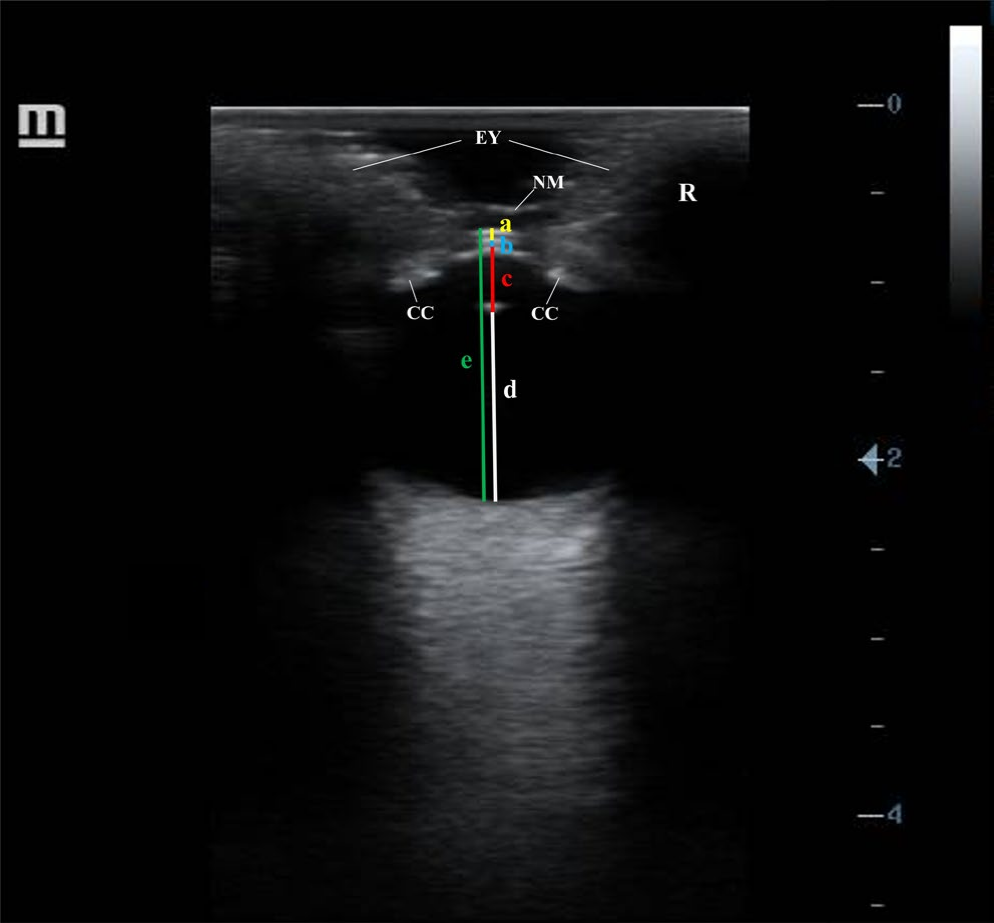
Ultrasonographic ocular biometry assessment in a Greater Caribbean manatee ( *Trichechus manatus manatus* ) calf in B‐mode employing a linear transducer set to 8.5 MHz. A = corneal thickness; B = anterior chamber depth; C = axial lens length; D = vitreous chamber depth; E = axial globe length; EY = eyelid; CC = ciliary body; NM = nictitating membrane; R = right eye.

Ocular biometry measurements were taken along the central optical axis of the eyeball at its maximum anteroposterior length. The following structures were measured (in centimeters): corneal thickness (CT), from the anterior to the posterior surface of the cornea; anterior chamber depth (ACD), from the posterior surface of the cornea to the anterior surface of the lens; axial lens length (ALL), from the anterior to the posterior surface of the lens; vitreous chamber depth (VCD), from the posterior surface of the lens to the back of the eyeball; and axial globe length (AGL), from the anterior corneal surface to the back of the eyeball (Figure 2).

### 2.3 Statistical Analyses

Statistical analyses were performed using the GraphPad Prism version 9.3 software for Windows (GraphPad Software Inc., San Diego, CA, USA). Results were expressed as means ± standard deviations (±SD). Data normalities were assessed by the Shapiro–Wilk test, and homogeneity of variances was evaluated by Bartlett's test. Non‐parametric tests were applied when data followed a non‐normal distribution (CT and ACD). All parameters were compared across different age categories by the Kruskal‐Wallis test followed by Dunn's multiple comparison test. For other biometric (ocular) parameters, comparisons between age categories were carried out through an analysis of variance (ANOVA), followed by Tukey's test. Spearman's correlation test was applied to investigate potential associations between manatee body and ocular biometric parameters, considering different age groups (calves, juveniles, and adults) as well as sex, using the “Analyze Data” procedure. An *α* = 0.05 significance level was adopted for all comparisons.

## 3 Results

Ocular ultrasound images were obtained employing the transcorneal approach, as the transpalpebral method did not allow for detailed eyeball visualization, preventing accurate biometric measurements. An oval eyeball shape was identifiable. The cornea appeared as a smooth, thin, convex double hyperechoic line with a hypo‐ to anechoic area between lines. The anterior chamber was shallow, and both the anterior and vitreous chambers were filled with homogeneous anechoic fluid. The lens exhibited a biconvex shape, positioned relatively forward in the eye, with smooth, thin, hyperechoic anterior and posterior surfaces and a homogeneous anechoic central portion. The ciliary body was visualized as a circular hyperechoic band located anterior to the lens equator. The nictitating membrane, when visible, appeared as a thin, slender, echogenic band over the corneal surface (Figure 2).

Regarding ocular parameters, the CT of both eyes did not vary significantly among age categories, with mean values ranging from 0.082 to 0.091 cm. The ACD, however, was significantly different between age groups. Right calf eyes recorded the lowest means (0.040 ± 0.005 cm), while higher values were observed in juveniles (0.051 ± 0.008 cm) and adults (0.052 ± 0.007 cm). The same pattern was observed in the left eye, although differences among age categories were less pronounced. The ALL also varied significantly between age groups, with calves exhibiting the lowest values (0.345 ± 0.028 cm) and adults, the highest (0.414 ± 0.020 cm) for both eyes. Conversely, no significant differences were found for VCD and AGL between age groups, indicating stable measurements among calves, juveniles, and adults (Table 2).

**TABLE 2:** Comparative analysis of the means (±SDs) of Greater Caribbean manatee ( *Trichechus manatus manatus* ) ocular parameters (*n* = 22), grouped by age category (calves, juveniles, and adults).

Variables	Overall (*n* = 22)	Calves (*n* = 7)	Juveniles (*n* = 8)	Adults (*n* = 7)
*Right eye*				
CT	0.087 ± 0.011	0.082 ± 0.012	0.088 ± 0.011	0.091 ± 0.010
ACD	0.048 ± 0.009	0.040 ± 0.005^B^	0.051 ± 0.008^A^	0.052 ± 0.007^A^
ALL	0.375 ± 0.035	0.345 ± 0.028^b^	0.367 ± 0.016^b^	0.414 ± 0.020^a^
VCD	1.151 ± 0.073	1.137 ± 0.076	1.155 ± 0.062	1.161 ± 0.089
AGL	1.663 ± 0.095	1.606 ± 0.098	1.663 ± 0.071	1.720 ± 0.094
*Left eye*				
CT	0.086 ± 0.011	0.082 ± 0.012	0.088 ± 0.012	0.087 ± 0.011
ACD	0.047 ± 0.008	0.041 ± 0.008^B^	0.048 ± 0.006^AB^	0.052 ± 0.007^A^
ALL	0.379 ± 0.037	0.345 ± 0.028^b^	0.381 ± 0.027^ab^	0.411 ± 0.025^a^
VCD	1.163 ± 0.068	1.147 ± 0.076	1.174 ± 0.045	1.166 ± 0.086
AGL	1.676 ± 0.091	1.617 ± 0.086	1.693 ± 0.062	1.717 ± 0.095

*Note:* Lowercase superscript letters (^a,b^) in the columns indicate significant differences between age categories (Tukey's test; *p* < 0.05). Uppercase superscript letters (^A,B^) indicate significant differences between age categories (Kruskal–Wallis test; *p* < 0.05).

Abbreviations: ACD, anterior chamber depth; AGL, axial globe length; ALL, axial lens length; CT, corneal thickness; VCD, vitreous chamber depth.

Positive and significant correlations between body and ocular biometric parameters were observed only in the calf group, namely between STL and the following ocular parameters: CT (*ρ* = 0.85; *p* < 0.0286), VCD (*ρ* = 0.82; *p* < 0.0302), and AGL (*ρ* = 0.85; *p* < 0.0238) for the right eye; and VCD (*ρ* = 0.82; *p* < 0.0302) and AGL (*ρ* = 0.85; *p* < 0.0238) for the left eye. No significant associations were identified between variables in the juvenile and adult groups, nor between BW and ocular parameters in any of the age groups (Table 3). Furthermore, no significant correlations were observed between body and ocular biometric parameters when males and females were analyzed separately within each age group.

**TABLE 3:** Significant correlations (*p* < 0.05) of Greater Caribbean manatees ( *Trichechus manatus manatus* ) between body and ocular parameters (*n* = 22), grouped per age category (calves, juveniles, and adults).

Variables	Ocular parameters									
	Right eye					Left eye				
	CT	ACD	ALL	VCD	AGL	CT	ACD	ALL	VCD	AGL
*Calves*										
BW	*ρ* = 0.75	*ρ* = −0.13	*ρ* = 0.21	*ρ* = 0.59	*ρ* = 0.67	*ρ* = 0.24	*ρ* = 0.02	*ρ* = 0.27	*ρ* = 0.59	*ρ* = 0.67
STL	*ρ* = 0.85^a^	*ρ* = −0.13	*ρ* = 0.43	*ρ* = 0.82^a^	*ρ* = 0.85^a^	*ρ* = 0.33	*ρ* = 0.09	*ρ* = 0.32	*ρ* = 0.82^a^	*ρ* = 0.85^a^
*Juveniles*										
BW	*ρ* = −0.30	*ρ* = −0.10	*ρ* = 0.09	*ρ* = 0.59	*ρ* = 0.52	*ρ* = −0.25	*ρ* = 0.02	*ρ* = 0.37	*ρ* = 0.27	*ρ* = 0.22
STL	*ρ* = −0.09	*ρ* = −0.27	*ρ* = −0.09	*ρ* = 0.61	*ρ* = 0.45	*ρ* = −0.06	*ρ* = −0.28	*ρ* = 0.30	*ρ* = 0.39	*ρ* = 0.27
*Adults*										
BW	*ρ* = 0.07	*ρ* = 0.23	*ρ* = 0.18	*ρ* = 0.44	*ρ* = 0.55	*ρ* = −0.45	*ρ* = 0.20	*ρ* = 0.33	*ρ* = 0.52	*ρ* = 0.37
STL	*ρ* = −0.03	*ρ* = 0.34	*ρ* = 0.30	*ρ* = 0.59	*ρ* = 0.70	*ρ* = −0.38	*ρ* = 0.40	*ρ* = 0.46	*ρ* = 0.66	*ρ* = 0.54

Abbreviations: ACD, anterior chamber depth; AGL, axial globe length; ALL, axial lens length; BW, body weight (kg); CT, corneal thickness; STL, straight total length (cm); VCD, vitreous chamber depth.

^a^ Significant correlations.

## 4 Discussion

Manatee strandings can be caused by both natural and anthropogenic factors, including vessel collisions, especially in areas with high vessel traffic and tourism, such as Florida, Belize, and Puerto Rico [25, 26, 27]. Other significant anthropogenic threats include habitat loss, fishing gear entanglement, and pollution [28, 29]. In Brazil, neonatal calves often become stranded after being separated from their mothers [1, 7, 30, 31]. The factors leading to these occurrences are probably associated with habitat loss, resulting in river siltation and hindering mothers’ ability to follow their calves [1, 26, 28, 29]. However, any health‐related aspects that could cause manatee strandings are still poorly understood. Previously reported ophthalmic alterations, such as corneal ulcers, keratitis and exophthalmia [11, 16, 20, 32], may comprise potential stranding causes, as vision is an important orientation sense for manatees [33, 34]. Thus, manatee ophthalmology assessments should be more deeply investigated in these events.

In this context, the ocular biometric data reported herein are paramount to support conservation strategies, especially in rehabilitation centers, serving as a technical and scientific basis for improving Greater Caribbean manatee public management, rehabilitation, and release policies. Such parameters can be incorporated into admission protocols, contributing to the identification of eye lesions, malformations, or structural and functional alterations associated with aging, as well as to pre‐release protocols, assisting in verifying visual manatee conditions.

The transcorneal technique applied in this study was proven effective for ocular ultrasonography in Greater Caribbean manatees, allowing for the acquisition of clear and detailed intraocular structure images. This approach was essential, as the transpalpebral technique was noted as unfeasible for the species, due to specific anatomical features like pronounced eyelid thickness and reduced palpebral fissure [12], that significantly hinder eyeball visualization. These findings differ from other marine animals, i.e., turtles [35], and terrestrial mammals like dogs and cats [36], cattle [37], and birds like *Amazona aestiva* [38], which possess relatively thin eyelids and greater eyeball exposure, allowing for the application of both transcorneal and transpalpebral techniques.

Manatee restraint for ocular ultrasonography assessments proved to be simple and safe, requiring only physical restraint without the need for any chemical control. This approach is feasible due to the docile behavior of the species, which contrasts with the handling of wild and more aggressive animals, such as felids, in which chemical restraint is essential to ensure both professional safety and wild animal welfare [39, 40]. Although physical restraint was sufficient, the use of topical ocular anesthetic was deemed necessary to provide manatee comfort and ensure their well‐being during examination. This type of local anesthesia is a recommended practice for all species, due to minimized ocular discomfort and ultrasonographic examination quality improvement [35].

Anatomically, the ultrasonographic evaluation of Greater Caribbean manatees revealed a small oval‐shaped eyeball (Right: 1.66 cm and Left: 1.67 cm), a convex cornea, a shallow anterior chamber, and a biconvex lens positioned relatively forward in the eye. These findings are consistent with anatomical descriptions reported for other sirenian species. Mass et al. [41], for example, described a small, nearly spherical eyeball (1.6 cm), a shallow anterior chamber, and an anteriorly positioned lens for the Florida manatee, whereas Piggins et al. [42] reported similar characteristics for the Amazonian manatee, with an eyeball diameter of 1.34 cm. The congruence of these structural features among species suggests that the ultrasonographic parameters established for the Greater Caribbean manatee may serve as a reference for assessing ocular morphology in other sirenians for which standardized ultrasonographic studies are not yet available.

The ocular biometric measurements obtained herein revealed important differences when compared to those of other species, both aquatic, such as sea turtles [35], and terrestrial, such as Jersey cattle [37]. Manatee corneal thickness is intermediate (0.09 cm), falling between the values recorded for sea turtles (0.04 cm) and cattle (0.17 cm). The anterior chamber depth is the smallest among the aforementioned species (0.05 cm) compared to 0.10 cm in sea turtles and 0.36 cm in cattle. Axial lens (0.38 cm) and eyeball (1.67 cm) lengths were also lower than those observed in cattle (1.92 and 3.27 cm, respectively), but similar to those reported for sea turtles (0.48 and 1.79 cm). Despite belonging to distinct taxonomic groups of mammals and reptiles, respectively, the similarities in eye measurements between manatees and sea turtles suggest that adaptations to a shared aquatic environment may have driven the development of comparable ocular structures [43]. On the other hand, although manatees and cattle share herbivorous feeding habits, the greater visual complexity demanded by terrestrial life may explain the presence of more developed ocular structures in cattle.

The significant anterior chamber depth differences noted among Greater Caribbean manatees' age groups indicate a continuous ocular development process. The lowest values were observed in calves, as expected, considering this age group's growth phase. The anterior chamber tends to deepen with development, closely linked to overall eyeball expansion and positively correlated with axial eye length increases [44].

The significant increase in axial lens length observed between calves and adults can be explained by the continuous growth of the lens throughout life, resulting from the deposition of new fibers in its equatorial region [45]. This progressive lens enlargement parallels overall ocular development and reflects the structural maturation required to meet the visual demands of adulthood [46, 47]. Underwater vision in marine mammals presents unique adaptations, as the refractive index of water is similar to that of corneal tissue, drastically reducing the cornea's refractive role. Under these conditions, the lens assumes a central role in both focusing and visual accommodation [43, 48, 49]. Thus, age‐related increases in axial lens lengths may represent an adaptive mechanism to optimize refractive power and focusing efficiency, ensuring effective visual performance during activities such as foraging, navigation, and social interactions.

Although manatee 19 was confirmed as an adult female based on age (7 years and 4 months), she exhibited lower body weight and total length compared to other adult manatees. Throughout her life, she was regularly evaluated for all routine hematological and pathological parameters at the institution, showing no clinically relevant alterations. All enclosures and the diet provided to this manatee were consistent with those of the other individuals at the institution, with no protocol changes. Therefore, the causes of this atypical body size remain unclear, with congenital alterations suspected. Ocular parameters were, however, consistent with those of the other adult manatees analyzed in the study. To assess whether this discrepancy could influence the results, statistical analyses were performed both including and excluding this individual, with no significant differences observed. The data were, consequently, retained in the sample, as her body condition may reflect natural individual variations without relevant implications for the biometric ocular parameters analyzed herein.

The positive correlations observed only in the calf group between straight total length and ocular parameters like corneal thickness, vitreous chamber depth, and axial globe length suggest that, at this early life stage, body and ocular development take place in a more synchronized and proportionate manner. Ultrasonographic studies in dogs of different ages have also demonstrated strong positive correlations between body weight and ocular parameters, such as equatorial lens length and axial globe length, supporting the idea of coordinated somatic and ocular growth during early life stages. These correlations in dogs tend to diminish with age, suggesting that this proportionality is more pronounced in puppies [50], reflecting ongoing ocular structure formation and maturation. On the other hand, the absence of significant correlations in juveniles and adults may indicate that ocular development reaches a maturation phase in which ocular parameters no longer vary proportionally with body size following body growth stabilization. This is consistent with studies on non‐human primates and humans, where ocular growth occurs quicker during early life stages and slower in adulthood, reflecting a differential developmental process between body systems [51, 52, 53].

No significant correlations were observed between body and biometric ocular parameters when males and females were analyzed separately within each age group. This may be due to the reduced sample size available for sex‐specific comparisons, which may have limited the statistical power to detect potential associations. On the other hand, it is also possible that sexual dimorphism does not play a relevant role in ocular biometric patterns in manatees. Further studies with larger sample sizes are, thus, required to clarify whether the absence of sex‐related differences reflects a biological characteristic of the species or results from current sampling limitations.

## 5 Conclusion

The use of ocular ultrasonography in the Greater Caribbean manatee was proven practical and feasible, with no harmful effects and allowing for indirect and real‐time intraocular structure visualization. Ocular biometric variables exhibited significant differences with increasing age in the species. These data are important in understanding manatee ocular system development and maturation, as well as in providing relevant conservation effort and health monitoring information across different manatee life stages in rehabilitation centers worldwide. Based on the findings reported herein, future studies should compare the ocular sonographic characteristics observed in the Greater Caribbean manatee to other manatee species considering different age ranges. Comparisons with the manatee's closest phylogenetic relative, the dugong ( *Dugong dugon* ) should also be carried out to determine the presence of shared findings or anatomical divergences. This approach could provide valuable information on the ocular anatomy evolution in sirenians and conservation and diagnostic imaging implications.

## Author Contributions

**Romário Parente dos Santos:** software, formal analysis, writing – review and editing, writing – original draft. **Fernanda Loffler Niemeyer Attademo:** investigation, writing – original draft, writing – review and editing, visualization, conceptualization. **Radan Elvis Matias de Oliveira:** conceptualization, investigation, writing – original draft, funding acquisition, methodology, validation, visualization, writing – review and editing, software, formal analysis, project administration, data curation, supervision, resources. **Antonio A. Mignucci‐Giannoni:** writing – original draft, writing – review and editing, investigation, formal analysis.

## Disclosure

The authors have not used AI to generate any part of the manuscript.

## Ethics Statement

This study was approved by the Animal Ethics Committee of the Federal Rural University of the Semi‐Arid Region (UFERSA) under approval number 31/2024. All activities carried out by both institutions were properly authorized by the relevant environmental agencies, according to permits issued by the Biodiversity Authorization and Information System (SISBIO), under permit numbers 13694‐12 for manatees at the Cetaceans of the Costa Branca Project at the Universidade do Estado do Rio Grande do Norte and 20685‐9 for manatees at the Aquatic Mammals Center of the Chico Mendes Institute for Biodiversity Conservation. Animals were not captured, restrained, sedated, or anesthetized solely for the purposes of this study.

## Conflicts of Interest

The authors declare no conflicts of interest.

## Data Availability

The data that support the findings of this study are available on request from the corresponding author. The data are not publicly available due to privacy or ethical restrictions.
